# The Relationship between Self-Efficacy and Psychosocial Care in Adolescents with Epilepsy

**DOI:** 10.1155/2015/756849

**Published:** 2015-12-31

**Authors:** Masoomeh Akbarbegloo, Leila Valizadeh, Vahid Zamanzadeh, Faranak Jabarzadeh

**Affiliations:** ^1^Department of Pediatric Nursing, Faculty of Nursing and Midwifery, Urmia University of Medical Sciences, Urmia 51389 47977, Iran; ^2^Tabriz University of Medical Sciences, Tabriz 51389 47977, Iran; ^3^Department of Pediatric Nursing, Faculty of Nursing and Midwifery, Tabriz University of Medical Sciences, Tabriz 51389 47977, Iran; ^4^Department of Medical Surgical Nursing, Faculty of Nursing and Midwifery, Tabriz University of Medical Sciences, Tabriz 51389 47977, Iran; ^5^Department of Medical Surgical Nursing, Student Research Committee, Faculty of Nursing and Midwifery, Tabriz University of Medical Sciences, Tabriz 51389 47977, Iran

## Abstract

*Introduction*. Studies about epilepsy are more associated with physiological aspects and drug therapy and far too little attention has been paid to psychological and social care, especially in teens. Hence, the present study aimed to assess relationship between self-efficacy and psychosocial care in adolescents with epilepsy.* Methods*. A cross-sectional association study was conducted on 74 consecutive adolescents aged 10 to 18 years with general attacks of epilepsy referred to Pediatric Neurology Clinics affiliated with the Tabriz University of Medical Sciences in 2013. Data were collected by interview using multisegment tools including demographic characteristics, self-efficacy scaling in children with epilepsy, and reporting tools for children psychosocial care.* Results*. Our study showed a significant association of self-efficacy with “information received” (*P* < 0.02) and also with “need for information or support” (*P* < 0.01) as well as “concerns and fears” (*P* < 0.01). The comments of doctor or nurse were directly associated with higher self-efficacy and patients' information needs were inversely associated with higher self-efficacy.* Conclusion*. For adolescents with epilepsy, providing educational materials such as pamphlets and booklets, designing especial websites, and setting especial meetings with and without parents separately are recommended. Scheduling psychosocial supports and collecting more information about this disorder for adolescents will be helpful.

## 1. Introduction 

Epilepsy influences all physical, psychological, and social aspects of affected individuals [1]. In Iran, 4.2 per 1000 school-age children suffered from epilepsy and about 56% of them belong to children and adolescents groups [2]. Caring of patients with epilepsy requires attention to all aspects of the illness as well as to teamwork between physicians, nurses, psychologists, 2 social workers, and other professionals [1]. In this regard, nurses can have effective advising and educating roles [3]. If one receives appropriate and timely caring services, the appearance of epilepsy related complications will perhaps be reduced or prevented [4]. children and adolescents with epilepsy have a multiplicity of physical, emotional, and cognitive concerns that could be addressed by a nurse in the outpatient clinic [5]. In total, comprehensive cares are recommended in epilepsy [6]. Moreover, the goal of treatment in health psychology is to change individuals' behaviors in a way that influences their response to a disease or illness. It is widely recognized that knowledge alone is not sufficient to change health behavior [7]. There is evidence that chronic disease self-management is influenced by an individual's beliefs about health, including self-efficacy [8]. Self-efficacy is a person's belief in his/her ability to successfully organize, control his/her health habit, and achieve valuable health outcomes. It is one aspect of individual motivation [7, 9]. The results of the studies on chronic disorders have shown that the individuals with high self-efficacy are more successful in management of self-care responsibilities, drug use, avoidance of stimuli, symptoms of disease, and control of health status [9–11]. So, self-efficacy is an effective factor on patients' ability to control the disease, coping with illness and drug control of epilepsy [12].

The needs in epileptic patients are widely varied including health protection needs (identifying different therapeutic and historical aspects of disease), barriers and safety (assessing disease-related risks such as possible risks of bathing time and also disease effects on the presence in the community), quality of care (quality of environment, the ability to manage the treatment, and the ability to perform life-saving drugs), specialized facilities (training, hosing, behavioral and psychological supports, and searching some diagnostic services such as MRI and EEG), and familial needs (emergency services, education, training, and support). The assessment by a nurse at the outpatient clinics could identify individual goals, specific concerns, and proper strategies to treat. It can be performed by interviewing, grading scales, and self-reporting questionnaires [5]. Conflict between the normal desire of the adolescent for independence and autonomy and the parental desire to maintain control is accentuated by the presence of epilepsy [13]. In addition, adolescence is associated with significant changes in mental, physical (pubertal), social, and psychological (identity, self-determination) aspects. Adolescents with chronic illnesses are at risk of various crises. The patients should be faced with complex evolutionary feature of this period and also stress induced by serious and prolonged treatment schedule. In adolescence, there is an effort to achieve independence, while these patients are conflicted because of compulsory reliance, surrendering, and loss of the sense of control. So, it is much harder to deal with illness for adolescents [13]. Improving self-efficacy might provide the foundation for long-term behavior change among adolescents with epilepsy by improving self-care skills which can lead to better control on disease. On the other hand, self-efficacy is an assurance that person can do a complementary behavior to achieve his desired goal [7]. For improving the self-efficacy, educating people about self-care is essential [14, 15]. One of the main factors affecting the outcome in patients with epilepsy is social cares. It has been demonstrated that the community support is not available for the social development of young people with epilepsy and thus they are susceptible to be picked on by others [6]. Also, patients with epilepsy are needed to assess social performance because they are more prone to social problems. One of the other factors that have important consequences for children with epilepsy is receiving information. If the patients have enough information about various aspects of the illness, returning to the normal living state like other people is possible [4]. In this regard, an important aspect of caring for children with epilepsy is educating people about the disease and providing emotional support for children and their family members [1]. The studies performed in western countries have shown that a large percentage of children with epilepsy felt the lack of information about their disease causing more concerns [16, 17]. Parents want to provide information to reduce the concerns of school, seizure, and the future of their children. Since the patient's needs can vary of the views of professionals and patients, and the evaluation of patient is critical in the planning of critical care processes [5]. Psychosocial cares include satisfaction of the total amount of information, attitudes of clinic staff, spending a time with the staff, and the comfort of being in contact with the staff [6]. Due to the changes in today's health care, this caring is increasingly presented ambulatory and evaluation of training, education, counseling, and support services run along with the evaluation and treatment of neurological and psychological assessments [5]. It should be noted that appropriate caring in an outpatient setting can also increase the self-efficacy of adolescents with chronic illnesses and this is because the expected positive correlation between self-efficacy and psychosocial care of adolescents suffered epilepsy with general attacks. Moreover, an extensive literature review did not result in finding research studies on the relationship between these two variables. Thus, the aim of this study was to investigate the relationship between self-efficacy and psychosocial care in outpatient clinics for adolescents with epilepsy.

## 2. Methods 

A cross-sectional association study was conducted on 74 consecutive adolescents aged 10 to 18 years with general attacks of epilepsy referred to Pediatric Neurology Clinics affiliated with the Tabriz University of Medical Sciences in 2013, within 6 months. This clinic is the main place for presenting neurology subspecialty cares to children in East Azerbaijan province. Inclusion criteria were using antiepileptic drugs and being with no history of concomitant physical impairments or mental retardation. The researchers obtained “baseline personal and social information” and administered the “Seizure Self-Efficacy Scale for Children (SSES-C)” and the “Child Report of Psychosocial Care.”

Seizure Self-Efficacy Scale for Children (SSES-C) was developed by Caplin et al. and used for assessing self-efficacy in children with epilepsy including some questions on confidence and belief in the patient ability to care as well as manage epilepsy. This tool is a 15-item scale that measures the degree of self-efficacy related to the management of the seizure disorder. Children rate each statement on a 5-point scale of 1 (*I'm very unsure I can do that*) to 5 (*I'm very sure I can do that*). The final score ranged between 15 and 75 with a higher score reflecting greater self-efficacy. Support for reliability and validity has been found [18].

Child Report of Psychosocial Care was firstly designed by Austin et al. including three parts. (a) The first part consisted of 6 items about receiving some information on illness by children that was expected to be provided by physician or nurse. Children rate each statement on a 3-point scale of 1 (less than what I wanted) to 3 (more than what I wanted) and the final score ranged between 6 and 18 with a higher score reflecting more information received by child. (b) The second part included 6 items in child's feelings about the occurrence of epileptic seizures and is based on 5-likert scaling from 1 (never) to 5 (more often). The total score of this statement ranged from 6 to 30 with a higher score reflecting more concern regarding epileptic attacks. (c) The third part included 8 items related to assessing the needs of children with epilepsy answered by “yes” or “no” with the final score ranging from 0 to 8 [16].

The original version of the questionnaire was translated from English to Persian; the accuracy of the translation was assessed by an expert in English language and literature. Then, the content validity of the questionnaire was determined through a panel of experts and also the reliability of the questionnaire was assessed by Cronbach's alpha coefficient through a study on a sample of 15 children. Cronbach's alpha coefficient was obtained: 0.85 for SSES-C, and also 0.81, 0.72, and 0.81 for the first, second, and third parts of the questionnaire for assessing child report of psychosocial cares, respectively.

The study was first approved by the Regional Committee for Medical Research Ethic at the Tabriz University of Medical Sciences. The two researchers conducted the study by referring to the clinic where eligible children for the study were selected. The details of the study were explained to the children and their parents and the written informed consent was obtained from all parents. Then, the data were gathered by personal interviews with all children. For statistical analysis, SPSS software (version 13.0) was employed. The mean ± SD or number (percentages) was used for describing children characteristics and also the level of self-efficacy and psychosocial cares. Association between self-efficacy and the level of receiving psychosocial cares was examined by Pearson's correlation test.

## 3. Results 

Eligible participants consisted of 74 children (40 girls and 34 boys) with ages 10 to 18 years (M = 12.72, SD = 2). The mean age of seizure onset was 7.48 years (SD = 3.4). Most participants (40%) had primary school education and 45% had secondary school education. The remainders were currently studying in high school. Positive epileptic history was in family of 27 percent. The major caregiver (93.2%) was both the mother and the father.

Most of the patients (76.7%) suffered mild epilepsy, 12.3% had moderate epilepsy, and others had severe epilepsy. The large majority of children (72.2%) had only one type of seizure (generalized tonic/colonic). With regard to the response to treatment, 21.9% of seizures were partially controlled, 6.8% were uncontrolled, and others were completely controlled. 21.65% of children had their epilepsy controlled with monotherapy and others required polypharmacy.

### 3.1. Self-Efficacy

The answers of adolescents to each option of self-efficacy questionnaire are presented in Table 1. As shown in this table, the highest level of reported self-efficacy was related to the item of “If there are problems about epilepsy, I can talk with my parents” (63.1%) and the lowest level was related to “When I am in school, I can predict and control my epilepsy” (11%). The mean score of self-efficacy was 45.4 ± 9.0 (95% CI: 43.3–47.4) totally.

### 3.2. Psychosocial Caring

Assessment of questions about receiving information from health personnel (doctors or nurses) showed that the mean score of this item was 10.0 ± 2.8 (95% CI: 9.4–10.7). More than half of the patients reported that received explanation was less than what they expected by doctor or nurse in all items except item “The doctors and nurses told me how the medicine worked” (Table 2).

### 3.3. Feelings and Concerns about Seizures

Assessment of questions in feelings and concerns about seizures and epileptic attacks obtained a mean score of 22.2 ± 5.7 (95% CI: 20.8–23.5) in this item. The highest concerns were related to “telling the state of seizure to others” (31.3%) and “avoid doing the things that the friends do due to attacks” (22.5%) (Table 3).

### 3.4. Educational Needs

Assessment of the questions on psychosocial cares in the part of educational needs indicated that the mean score of subjects was 6.1 ± 2.1 (95% CI: 5.6–6.6). As summarized in Table 4, adolescents felt the need to get more information.

The study of association between self-efficacy and three dimensions of psychosocial caring using Pearson's correlation test showed that the score of self-efficacy in adolescents was directly associated with receiving more information and inversely associated with feelings and concerns about seizures and educational needs (Table 5). On the other hand, by raising awareness of epilepsy, reducing negative feelings about epilepsy, and reducing educational needs of adolescents, their self-efficacy on controlling epilepsy can be effectively increased.

## 4. Discussion

Appropriate management of chronic disorders is directly associated with professional supports, proper outpatient cares, and the patient's belief system. Perceived self-efficacy has become an important and useful construct in psychology because it is related to the willingness and the ability of people to engage in various behavioral challenges including preventive and disease management behaviors [19]. Individuals who provide social support experience less depression, heightened self-esteem and self-efficacy, and improved quality of life, even after adjusting for baseline health status and socioeconomic status [20]. Therefore, the present study attempted to determine the association between self-efficacy and the level of psychosocial cares in teenagers with epilepsy in a pediatrics neurology outpatient clinic. In this study, self-efficacy was significantly associated with explanations of health personnel, concerns about epilepsy state, and also educational needs so that higher level of information given by the physician as well as lower needing information was related to higher self-efficacy. In fact, by increasing the level of information, self-efficacy was increased in parallel. In order to increase motivation following the increase of efficacy, Bandura believed that people with high self-efficacy are more motivated and do more attempts to overcome the challenges of life [21, 22]. With regard to the attempt to increase social support following the increase of self-efficacy, Videbeck thought that those with high self-efficacy are seeking support from others [23]. Pajares also believed that people with low self-efficacy refrain from discussing complex issues and problems and thus are unable to achieve their goals, select introspection rather than remedy the problem, and focus on their weaknesses and barriers when dealing with stress, and in total they are lodged in the problem and suffered depression. In contrast, those with high self-efficacy used some challenge to solve it when faced with problems and do not perceive it as a threat [24]. Studies have indicated that more confidence in ability to perform self-care behaviors can lead to more tendency to perform the desired behaviors [25, 26]. Adams et al. believed that psychosocial problems should be identified and early shown. Giving simple and reliable information is very helpful to cope with the feelings of inferiority and shyness [27]. In a study by Baker et al. in England to assess the psychosocial effects of epilepsy in adolescents, it was indicated that repeated seizures are associated with lower self-efficacy and also tonic-colonic seizure was associated with appearance of depression [28]. In another study by Frizzell and colleagues, it was shown that holding training sessions in epilepsy syndrome and epilepsy effects on lifestyle resulted in the increase of knowledge and self-efficacy in adolescents compared with before intervention. They also indicated, by educational intervention, even without psychological approaches, achieving increased level of psychological functioning [29]. In another study by Zamnzadeh et al., following educational sessions in diabetics, the rate of self-efficacy in part of managing psychosocial aspects was significantly increased [30]. Baker et al. also showed that lower knowledge was in line with lower self-efficacy as well as higher level of depression [28]. Talking with physicians and participating in decision-making is an aspect of communication that contribute to tend from asking the questions to action. Sense of satisfaction of caregivers and getting support cause a sense of competence and self-efficacy, leading to better control of epilepsy [12]. Although availability can be helpful, developing confidence is based more on verbal emphasis and encouragements [21]. Creating an environment where patients feel comfortable to discuss issues related to living with epilepsy and also supplying and providing supportive statements and sentences that respect the individual's ability to manage are important in the creation of trust. In addition, the nurses and doctors often emphasize their previous attempts at self-management, including the administration of drugs, creation of a safe environment, and centrally monitored seizures [31]. The results of the study by DiIorio et al. have shown that providers should consider social and emotional aspects of epilepsy that involve in providing confidence for the daily management of epilepsy [31].

## 5. Conclusion

The strong expressed need for information about handling future seizures and the need to talk about strategies for dealing with seizures that occur at school indicate that these might be areas to include in an intervention that aimed at enhancing self-efficacy [32].

## Figures and Tables

**Table 1:** Self-efficacy in epileptic patients aged 10 to 18 years.

SSES-C	No answer	Not at all	Nearly sure	Doubtfully	Partially sure	Pretty sure
I can talk with my parents about problems of epilepsy	1	4 (5.5)	3 (4.1)	5 (6.8)	15 (20.5)	46 (63.1)
I can stop myself from doing things that will aggravate epilepsy	1	6 (8.2)	6 (8.2)	7 (9.6)	12 (16.4)	42 (57.6)
I can do things the doctor said to control epilepsy	1	2 (2.7)	4 (5.5)	6 (8.2)	20 (27.4)	41 (56.2)
I can check the status of my seizures by avoiding the things that make it worse	1	2 (2.7)	8 (11.0)	4 (5.5)	23 (31.5)	36 (49.3)
I can talk with the doctor or nurse when you have questions about epilepsy	1	9 (12.4)	3 (4.1)	6 (8.2)	20 (27.4)	35 (47.9)
I can control my seizures by selecting appropriate activities	0	5 (6.8)	5 (6.8)	11 (14.8)	20 (27.0)	33 (44.6)
I can control my epilepsy so can participate easily in school-related activities	0	0 (0.0)	6 (8.2)	4 (5.5)	28 (38.4)	35 (47.9)
I can control my epilepsy situation by refraining from doing things that make it worse	0	3 (4.1)	7 (9.6)	6 (8.2)	22 (30.2)	35 (47.9)
I can control my epileptic condition because I can handle all the problems that it creates epilepsy	0	2 (2.8)	5 (6.8)	13 (17.8)	24 (32.9)	29 (39.7)
I can control my seizures despite some troubling issues in my family	1	14 (19.2)	9 (12.3)	12 (16.4)	24 (32.9)	14 (19.2)
I can predict and control their epilepsy when I'm at school	4	40 (57.2)	5 (7.1)	8 (11.4)	6 (8.6)	11 (15.7)
I can control my seizures even when I am angry or sad	1	29 (39.2)	6 (8.2)	9 (12.3)	18 (24.7)	11 (15.1)

**Table 2:** Child report of psychosocial care subscale 1 (patient received explanation from doctor or nurse).

Psychosocial care subscale 1	Less than I wanted (percent)	Just as much as I wanted (percent)	More than I wanted (percent)
The doctors and nurses told me what to do if I felt an attack coming on.	53 (71.6%)	16 (21.6%)	5 (6.8%)
The doctors and nurses talked to me about my fears and worries about my seizure condition.	51 (68.9%)	20 (27%)	3 (4.1%)
The doctors and nurses told me about possible problems or side effects with the medicine.	49 (67.1%)	21 (28.8%)	3 (4.1%)
I have had a chance to ask questions about my seizure condition.	46 (62.2%)	24 (32.4%)	4 (5.4%)
The doctors and nurses explained my seizure condition to me.	45 (60.8%)	23 (31.1%)	6 (8.1%)
The doctors and nurses told me things I can and can not do because of seizures.	39 (54.2%)	24 (33.3%)	9 (12.5%)
The doctors and nurses told me how the medicine worked.	36 (48.6%)	31 (41.9%)	7 (9.5%)

**Table 3:** Child report of psychosocial care subscale 2 (feelings and concerns about seizures).

Psychosocial care subscale 2	Never (percent)	Not often (percent)	Sometimes (percent)	Often (percent)	Very often (percent)
How often do you worry about telling others about your seizure condition?	29 (39.2%)	11 (14.9%)	6 (8.1%)	5 (6.8%)	23 (31%)
How often do you avoid doing something with your friends because of fear about having a seizure attack?	29 (40.8%)	11 (15.5%)	11 (15.5%)	4 (5.6%)	16 (22.6%)
How often do you worry about having another seizure attack?	37 (50%)	8 (10.8%)	14 (18.9%)	2 (2.7%)	13 (17.6%)
How often are you worried about what others will say about your seizure condition?	38 (51.4%)	10 (13.5%)	11 (14.9%)	2 (2.7%)	13 (17.6%)
How often do you worry about being sick because of the seizure condition?	36 (48.6%)	13 (17.6%)	8 (10.8%)	5 (6.8%)	12 (16.2%)
How often do you worry about hurting yourself because of a seizure attack?	36 (48.6%)	19 (25.7%)	10 (13.5%)	3 (4.1%)	6 (8.1%)

**Table 4:** Child report of psychosocial care subscale 3 (educational needs).

Psychosocial care subscale 3 (ranked)	Yes number (percent)	No number (percent)
More information about any activities or things you can or cannot do because of seizures?	68 (91.9%)	6 (8.1%)
More information about keeping safe during a seizure? miss = 1 (1.4%)	65 (89%)	8 (11%)
More information about how to handle future seizures?	62 (83.8%)	12 (16.2%)
More information about your seizure condition?	60 (81.1%)	14 (18.9%)
More information about possible causes of your seizure condition?	60 (81.8%)	14 (18.2%)
More information about your medication?	59 (79.7%)	15 (20.3%)
To talk to someone about how to handle seizures at school?	41 (55.4%)	33 (44.6%)
To talk to someone about how your seizure condition might affect your future?	40 (54.1%)	34 (45.9%)

**Table 5:** Association between psychosocial cares and self-efficacy in adolescents with epilepsy.

Variable	Pearson's coefficient	*P* value
Self-efficacy/comments on physician or nurse	0.25	0.022
Self-efficacy/concern about epilepsy state	−0.27	0.016
Self-efficacy/educational needing	−0.31	0.006
